# Quantification of Optic Disc Edema during Exposure to High Altitude Shows No Correlation to Acute Mountain Sickness

**DOI:** 10.1371/journal.pone.0027022

**Published:** 2011-11-01

**Authors:** Gabriel Willmann, M. Dominik Fischer, Andreas Schatz, Kai Schommer, Andre Messias, Eberhart Zrenner, Karl U. Bartz-Schmidt, Florian Gekeler

**Affiliations:** 1 Centre for Ophthalmology, University of Tuebingen, Tübingen, Baden-Württemberg, Germany; 2 Department of Sports Medicine, Medical Clinic, University Hospital Heidelberg, Heidelberg, Germany; 3 Department of Ophthalmology, Otorhinolaryngology and Head & Neck Surgery, School of Medicine of Ribeirão Preto, University of São Paulo, São Paulo, Brazil; Institut de la Vision, France

## Abstract

**Background:**

The study aimed to quantify changes of the optic nerve head (ONH) during exposure to high altitude and to assess a correlation with acute mountain sickness (AMS). This work is related to the Tuebingen High Altitude Ophthalmology (THAO) study.

**Methodology/Principal Findings:**

A confocal scanning laser ophthalmoscope (cSLO, Heidelberg Retina Tomograph, HRT3®) was used to quantify changes at the ONH in 18 healthy participants before, during and after rapid ascent to high altitude (4559 m). Slitlamp biomicroscopy was used for clinical optic disc evaluation; AMS was assessed with Lake Louise (LL) and AMS-cerebral (AMS-c) scores; oxygen saturation (SpO_2_) and heart rate (HR) were monitored. These parameters were used to correlate with changes at the ONH. After the first night spent at high altitude, incidence of AMS was 55% and presence of clinical optic disc edema (ODE) 79%. Key stereometric parameters of the HRT3® used to describe ODE (mean retinal nerve fiber layer [RNFL] thickness, RNFL cross sectional area, optic disc rim volume and maximum contour elevation) changed significantly at high altitude compared to baseline (*p*<0.05) and were consistent with clinically described ODE. All changes were reversible in all participants after descent. There was no significant correlation between parameters of ODE and AMS, SpO_2_ or HR.

**Conclusions/Significance:**

Exposure to high altitude leads to reversible ODE in the majority of healthy subjects. However, these changes did not correlate with AMS or basic physiologic parameters such as SpO_2_ and HR. For the first time, a quantitative approach has been used to assess these changes during acute, non-acclimatized high altitude exposure. In conclusion, ODE presents a reaction of the body to high altitude exposure unrelated to AMS.

## Introduction

Swelling of the optic nerve head (or optic disc edema, ODE) at high altitude was first described in 1969 in Indian soldiers stationed between 3300–5500 m above sea level [1]. In this study, most affected soldiers also presented with signs of high altitude cerebral edema (HACE) such as severe headache, stupor, coma, and increased intra-cranial pressure (ICP). Optic disc edema has since been reported by several other authors in mountaineers with HACE [2], [3] and in case reports where it was found in conjunction with high altitude retinopathy, a clinical entity of hemorrhages and other retinal alterations [4], [5], [6], [7], [8]. In 3 out of 21 climbers who had ascended to altitudes above 7500 m Wiedmann and Tabin described persistent ODE [9]. The most recent study by Bosch et al. funduscopically reported ODE in climbers at very high altitude [10]. Moreover, they described a positive and statistically significant correlation of their findings with AMS.

The term AMS describes an altitude-related potentially life threatening condition with headache as the main clinical characteristic [11]. Associated vegetative symptoms include nausea, insomnia, anorexia and dizziness. Although generally benign, AMS can progress to HACE, which is life threatening if left untreated [12]. Underlying mechanisms of AMS are still under debate with some research groups favoring cerebral edema of varying degree as the underlying mechanism for all forms of the disease [13]. In addition, indirect evidence suggests brain edema as a possible cause of AMS [14], [15]. However, another study measuring intracranial pressure by lumbar puncture during hypoxia exposure was not able to detect significant differences in subjects with or without AMS [16]. Thus pathophysiology of AMS remains a conundrum.

Examination of the optic nerve head in the posterior segment of the eye provides a unique possibility to directly and non-invasively visualize the state of the cranial nerve. Causes for alterations of the optic nerve head can be manifold and may point to various underlying diseases and pathophysiologic mechanisms, including toxic, inflammatory and infectious diseases, compression, ischemia and also to high ICP due to increased cerebro-spinal fluid volume or cerebral edema [17]. A positive correlation of AMS and ODE may therefore support a causative role of high ICP in patients with AMS.

This study was undertaken to objectively quantify optic disc morphology using state of the art technology by using confocal scanning laser ophthalmoscopy (cSLO) in addition to stereoscopic slit-lamp funduscopy in a defined setting in volunteers exposed to high altitude at the Capanna Margherita (4559 m, Italy). AMS was assessed by clinical questionnaires and correlation between quantitatively measured ODE and AMS was tested.

## Methods

### Study design

**Figure 1:**
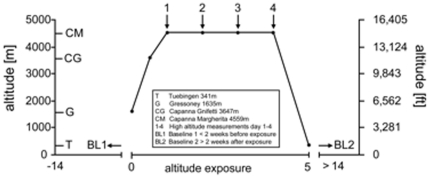
Ascent profile to the Capanna Margherita (4559 m). Ascent profile of participants to the Capanna Margherita (4559 m). Numbers indicate the four HRT3® measurements performed at high altitude.

 In this prospective field study 18 participants ascended to the Capanna Margherita (CM; Valais Alps, Italy) accompanied by a mountain guide according to the following ascent profile (Figure 1): day0 from Gressoney (Italy) 1635 m to Punta Indren 3260 m by cable car followed by 2 hours (hrs) of hiking to the Capanna Gnifetti 3647 m; day1 ascent to the CM 4559 m in 4–6 hrs. All participants ascended within 24 hrs from Gressoney to the CM. Fourteen participants spent 3 nights (from day1 to day4) and 4 participants (investigators) 12 nights at the CM before descending back to Gressoney in one day. Prior to baseline recordings (BL1 = before and BL2 = after the expedition) and high altitude exposure, subjects had to spend >14 days below 2000 m and were not allowed to climb above until the start of the research expedition. Included were healthy, physically fit participants (7 females and 11 males; age 25–54 years, mean = 35±8 (SD) years) capable of climbing to the CM within 24 hrs from Gressoney. General exclusion criteria were any type of cardiac or respiratory disease. None of the participants had a history of HACE or high altitude pulmonary edema (HAPE). Only non-steroidal anti-inflammatory agents were allowed and administered after consultation of a study group physician. Ophthalmological exclusion criteria were refractive error greater than ±5 diopters spherical equivalent, astigmatism more than ±2 diopters, presence of disorders affecting the optic disc, or optical opacities limiting imaging quality. Due to set-up procedures of the equipment at high altitude 14 participants were measured on day1, 16 participants on day2 and day3 and all 18 participants on day4. The 4 participants, who spent 13 days at high altitude, were measured regularly over time.

The study was performed in accordance with the tenets of the Declaration of Helsinki 1975 (1983 revision) and was approved by the local IRB (Ethik-Kommission der Medizinischen Fakultät und am Universitätsklinikum Tuebingen, IEC project number: 258/2010B01). All participants gave written informed consent after having been informed of the nature of the research expedition.

### Clinical assessment of Acute Mountain Sickness and vital parameters

For assessment of AMS the Lake-Louise (LL) and the AMS-cerebral score (AMS-c) of the Environmental Symptom Questionnaire (ESQ III) were used once at BL1 and BL2, and twice daily during altitude exposure [18], [19]. All subjects were thoroughly informed regarding the nature of the questionnaires prior to baseline recordings. The same study staff was involved at all time points and was available for help in completing the questionnaires on as-needed basis. AMS was assumed when both scores met the cut-off criteria set for LL ≥5 in the presence of a headache and AMS-c ≥0.70 as described previously [16]. Measurements of oxygen saturation and HR were performed once at BL1 and BL2, at high altitude in the morning before getting up and in the evening after >5 minutes rest with a finger pulse oximeter (oxy control 4c®, Geratherm Medical AG, Geschwenda, Germany). Values were recorded after one minute of steady measurement. Blood hemoglobin content (Hb) and hematocrit were measured from samples drawn from an antecubital vein through the University Hospital's laboratory at BL1 and BL2. At high altitude whole blood was again drawn from an antecubital vein and collected in EDTA containing tubes (Monovette, Sarstedt, Nümbrecht, Germany) for measurements of Hb using an Hb-miniphotometer® (Dr. Bruno Lange GmbH, Berlin, Germany) and for measurements of hematocrit following a centrifugation at 6 min of 13 000×g (micro-hematocrit centrifuge, Hettich, Tuttlingen, Germany) on day1, day2, and day4 at high altitude.

### Slit lamp biomicroscopy and quantitative analysis of optic nerve head

All participants underwent general ophthalmological examination including best corrected visual acuity and slit lamp stereo biomicroscopy of the anterior and posterior segment. According to the criteria for detecting ODE described by Walsh and Hoyt, the right and left optic discs were judged by the same experienced ophthalmologist either as swollen, not swollen or equivocal once at BL1 and BL2, and daily during high altitude exposure [20] using a LED-powered slit lamp (BQ900®, Haag-Streit International, Koeniz, Switzerland) with non-contact wide field and high mag lenses (Digital Wide Field® and Digital High Mag®, Volk Optical Inc., Mentor, OH, USA). All equipment was transported by helicopter (Air Zermatt AG Heliport, Zermatt, Switzerland) in airfreight containers provided by the manufacturers.

To quantitatively measure the changes of the ONH two identical HRT3® (Heidelberg Engineering, Heidelberg, Germany) devices were used for baseline measurements and for measurements at altitude [21]. To exclude systemic error and ensure comparability of measurements, we exported the raw data from baseline recordings and imported it before follow-up measurements at altitude took place to correct for errors such as tilt and orientation. Calibration of HRT3® devices was performed before each measurement according to instructions by the manufacturer to ensure comparability of measurements. Details of the instrument and its reproducibility have been published elsewhere [22], [23], [24]. Briefly, it features a 670-nm light source in a confocal optic pathway that acquires topographic images as a series of 32 two-dimensional transverse optic sections (each 256×256 pixels), acquired in ca. 1.5 seconds. To correct for magnification errors, patients's keratometry readings were considered by the software. Each recording consisted of the mean of three consecutive scans; measurements were performed at baseline once before (BL1), daily during and once again after exposure (BL2) to high altitude on both eyes. After the first measurement at BL1 an optic disc contour line was drawn manually along the inner margin of the peripapillary scleral ring by the same experienced operator. All consecutive measurements were automatically corrected for orientation/tilt and fitted according to the baseline measurement to allow optimal follow-up calculation. The built-in HRT software version 2.01 calculated the stereometric parameters of the optic nerve head. For evaluation of changes at the ONH, all stereometric parameters from all measurements of both eyes were exported for statistical analysis as tab-delimited text files. As measurements of the left and right eye revealed no significant differences (see results section), only the right eye was considered. The following 12 stereometric parameters were used for further statistical analysis: mean RNFL thickness, rim area, RNFL cross sectional area, rim volume, average variability, cup area, cup volume, disc area, maximum contour elevation, maximum cup depth, mean cup depth, and vertical cup/disk ratio. They were selected for their potential to detect ODE and to test general validity of the recordings. To assess a potential correlation of ODE with AMS, the following stereometric parameters were correlated with AMS-c, LL, SpO_2_, HR, and age: rim volume, mean RNFL thickness, RNFL cross sectional area and maximum contour elevation; all quantitative measurements were performed in the morning and recordings of day2 were used for correlation analysis.

### Statistical Methods

For statistical analysis JMP® was used (Version 8.0.2, SAS Institute, Cary, NC). Comparisons were performed by multivariate analysis of variance (MANOVA) for repeated measures. Data are shown in terms of intra-individual ratios (value during exposure/value at BL1) and the 95% confidence interval for each time point. Additional analysis of the data using the paired t-test for each time point vs. BL1 resulted in equal outcomes. To evaluate a possible correlation between changes at the ONH and AMS or basic physiologic parameters the Pearson's correlation coefficient between recorded HRT3® values and clinical parameters (SpO_2_, HR, AMS-c, LL and age) was calculated.

## Results

All 18 participants reached the CM using the described ascent profile and completed all planned examinations during 4 days at high altitude as noted in Figure 1. Incidence of AMS was 61% in the evening after arrival at the CM on day1, 55% in the morning of day2 and decreased distinctly thereafter with a tendency for higher values in the mornings (for details of AMS scores see Table 1). The maximum LL recorded during our high altitude stay was 13 on day1; the top AMS-c score was reached in the morning of day3 with 2.79 in one participant, who had to be treated with 4 mg dexamethasone intravenously on day3 and again orally on day4 before descent. AMS-c, LL and overall well-being improved rapidly after initial steroid treatment. Mean LL was highest in the evening of day1 (5.7±3.1; mean ± SD) and AMS-c was highest in the morning of day2 (1.13±0.75). Heart rate was increased in all participants at high altitude, while oxygen saturation was expectably decreased; hematocrit and hemoglobin values did not change significantly during high altitude exposure (see Table 1).

**Table 1:** Overview of systemic parameters of THAO study participants.

	BL1	day1 evening	day2 morning	day2 evening	day3 morning	day3 evening	day4 morning	BL2
LL	0	5.70±3.10	5.22±2.58	3.83±2.04	4.06±3.04	2.06±1.40	2.28±1.90	0
AMS-c	0	1.05±0.67	1.13±0.75	0.66±0.61	0.65±0.72	0.22±0.30	0.30±0.32	0
AMS [%]	0	61	56	28	28	0	11	0
Heart rate (HR) [min^−1^]	60.33±7.22	89.40±5.92	82.73±9.71	80.06±11.72	76.78±11.39	75.39±13.19	72.22±12.73	58.78±6.38
SpO2 [%]	98.50±1.29	70.60±5.18	73.00±6.01	74.28±6.61	74.22±5.62	77.28±3.82	79.56±5.15	98.50±1.25
Hemoglobin (Hb) [g/dL]	14.32±1.29	13.65±1.10	14.21±1.18	N/A	N/A	N/A	14.97±1.09	15.78±0.75
Hematocrit (Hct) [%]	42.17±3.17	43.19±2.37	44.97±3.24	N/A	N/A	N/A	45.29±1.98	44.06±3.24

n = 18; Hb and Hct for 1 evening and 2 morning n = 16; values are given as mean±standard deviation; N/A = not applicable.

Incidence of ODE as assessed by slit-lamp biomicroscopy was 0% at BL1 and reached 64% on day1 (evening) at the CM; incidence increased to 79% on day2 and decreased to 64% on day3 (no slit lamp biomicroscopy was performed on day4 due to organization of descent). At BL2 all discs had returned to normal and no ODE was observed. Data are given in Table 2. Optic discs judged as equivocal were not considered. Interestingly, optic disc margins were blurred most noticeably on the nasal side of the disc.

**Table 2:** Overview of clinical funduscopic results.

	BL1	day1	day2	day3	BL2
swollen	0	9	11	10	0
equivocal	0	2	1	2	0
not swollen	14	3	2	2	14
optic disc edema [%]	0	64	79	64	0

n = 14.

**Figure 2:**
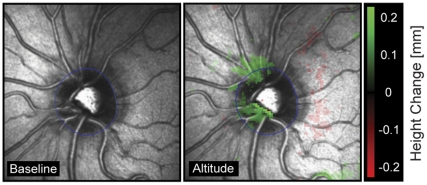
Automated Track Change Analysis (TCA) of the optic nerve head. **Automated** Track Change Analysis (TCA) of the optic nerve head shows increase in neuroretinal rim height in a representative participant between baseline recording (BL1, left) and recording at high altitude (day2, right). The change is color coded with green indicating an increase of tissue (see scale on the right).

**Figure 3:**
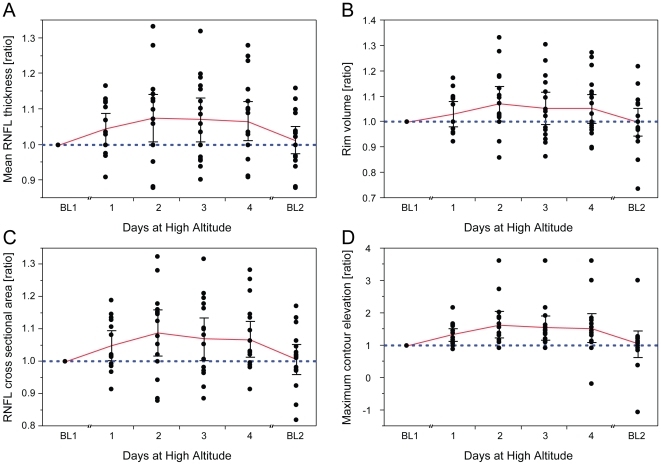
Changes of key stereometric parameters at high altitude. Intraindividual changes (expressed as ratios) of key stereometric parameters at altitude show statistically significant but reversible optic disc edema at high altitude. (A) Mean retinal nerve fiber layer (RNFL) thickness, (B) rim volume, (C) RNFL cross sectional area and (D) maximum contour elevation are significantly increased between day2 and day4 of altitude exposure (*p*<0.05). Individual measurements at baseline prior to the climb (BL1) were normalized to display intraindividual ratios of values at high altitude/BL1. Blue dashed lines indicate BL1 level; red lines indicate mean; whiskers indicate 95% confidence interval; number of subjects: n_day1_ = 14, n_day2–3_ = 16, n_day4_ = 18.

Statistical analysis of quantitative stereometric parameters (HRT3®) confirmed the clinical impression of symmetric ODE changes with no statistically significant differences between the right and left eye for BL1, BL2 and any of the high altitude recordings of day1–4 (data not shown). Therefore, only measurements of right eyes are presented in the following. Quantitative measurements (HRT3®) before and after high altitude exposure (BL1 *vs*. BL2) did not reveal statistically significant differences (95% confidence interval (CI) ; *p*>0.50) for all 12 outcome measures (see methods section and Table S1), indicating complete reversibility of all changes that had developed during exposure to high altitude. Specifically, CI and *p*-values (BL1 *vs*. BL2) of all key stereometric parameters were: rim volume (CI for BL1 = 0.33 to 0.47 and BL2 = 0.33 to 0.46; *p* = 0.92), mean RNFL thickness (CI for BL1 = 0.24 to 0.30 and BL2 = 0.24 to 0.30; p = 0.90), RNFL cross sectional area (CI for BL1 = 1.13 to 1.45 and BL2 = 1.13 to 1.45; *p* = 0.99) and maximum contour elevation (CI for BL1 = −0.16 to −0.09 and BL2 = −0.17 to −0.11; *p* = 0.60). The complete data set is provided as Table S1. As illustrated in Figure 2, HRT3® analysis using the automated track change analysis (TCA) showed an increase in neuroretinal rim height between BL1 and recording at high altitude (day2). However, six hrs after arrival at the CM on day1 the increase in RNFL thickness was not statistically significant, but reached significance in the morning at day2, 3 and 4 (Figure 3A). An identical pattern was seen for the ratios of rim volume, RNFL cross sectional area and maximum contour elevation, that were also significantly higher on day2, 3 and 4 than at BL1, but not on day1 (Figure 3B–D). Average variability, maximum and mean cup depth remained stable overall, but were significantly increased on day1 or on day2 respectively. Cup volume, cup and disk rim areas, and vertical cup/disk ratio remained unchanged at altitude. Data of all 12 stereometric parameters at high altitude from day1–4 compared to BL1 recordings are summarized in Table 3.

**Table 3:** Overview of selected HRT3® stereometric parameters for all days measured.

	day1	day2	day3	day4	BL2
**average variability (SD) [mm]**	**1.27**±**0.67 (1.03–1.51)** *	1.14±0.43 (0.92–1.37)	1.20±0.60 (0.98–1.43)	1.00±0.35 (0.79–1.21)	1.08±0.43 (0.87–1.29)
cup area [mm^2^]	1.10±0.40 (0.94–1.26)	1.10±0.39 (0.95–1.25)	1.03±0.42 (0.88–1.18)	1.01±0.25 (0.87–1.15)	1.04±0.16 (0.90–1.18)
cup volume [mm^3^]	1.09±0.36 (0.93–1.25)	1.11±0.35 (0.96–1.25)	0.94±0.36 (0.80–1.09)	0.99±0.24 (0.85–1.13)	1.06±0.32 (0.92–1.19)
disc area [mm^2^]	1.00±0.00 (0.99–1.01)	1.00±0.00 (0.99–1.01)	1.00±0.00 (0.99–1.01)	1.00±0.00 (0.99–1.01)	1.00±0.00 (0.99–1.01)
**maximum contour elevation [mm]**	1.30±0.30 (1.00–1.61)	**1.57±0.65 (1.28–1.85)** *	**1.48±0.60 (1.20–1.77)** *	**1.48±0.75 (1.21–1.74)** *	1.06±0.71 (0.79–1.33)
**maximum cup depth [mm]**	1.02±0.08 (0.98–1.07)	**1.05±0.09 (1.01–1.09)** *	1.00±0.12 (0.96–1.04)	1.01±0.09 (0.97–1.04)	1.01±0.05 (0.97–1.05)
**mean cup depth [mm]**	1.05±0.15 (0.99–1.11)	**1.06±0.12 (1.01–1.11)** *	0.99±0.14 (0.93–1.04)	0.99±0.12 (0.94–1.05)	1.01±0.07 (0.96–1.07)
**mean RNFL thickness [mm]**	1.05±0.08 (1.00–1.10)	**1.08±0.13 (1.03–1.12)** *	**1.07±0.12 (1.03–1.12)** *	**1.07±0.11 (1.02–1.11)** *	1.01±0.08 (0.97–1.06)
rim area [mm^2^]	1.00±0.03 (0.98–1.02)	1.00±0.04 (0.98–1.02)	1.02±0.04 (1.00–1.03)	1.01±0.03 (1.00–1.03)	1.00±0.05 (0.98–1.01)
**rim volume [mm^3^]**	1.03±0.09 (0.98–1.09)	**1.08±0.14 (1.03–1.14)** *	**1.06±0.13 (1.00–1.12)** *	**1.06±0.13 (1.01–1.11)** *	1.00±0.12 (0.95–1.05)
**RNFL cross sectional area [mm^2^]**	1.05±0.07 (1.00–1.10)	**1.08±0.12 (1.04–1.13)** *	**1.07±0.11 (1.02–1.11)** *	**1.06±0.10 (1.02–1.11)** *	1.01±0.08 (0.96–1.05)
vertical cup/disk ratio	0.96±0.10 (0.86–1.06)	0.99±0.25 (0.90–1.09)	0.94±0.23 (0.84–1.04)	0.94±0.22 (0.85–1.039	0.99±0.19 (0.90–1.08)

*p-value < 0.05; bold indicates significant change in stereometric parameter; values are given as mean ratios ± standard deviation (95% confidence interval).

**Figure 4:**
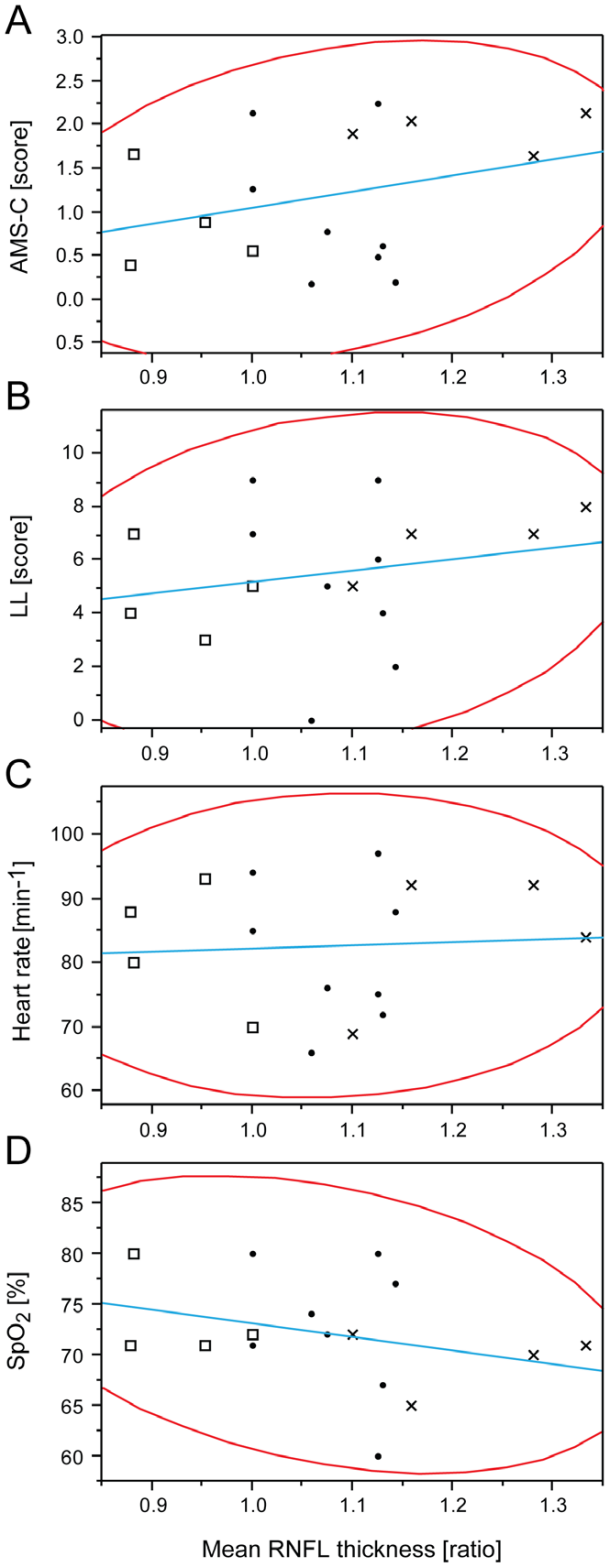
Correlation of optic disc edema with AMS and clinical parameters during high altitude exposure. Optic disc edema does not correlate with AMS-c, LL, HR or SpO_2_. Representative correlation analyses between mean retinal nerve fiber layer (RNFL) thickness ratios and (A) AMS-c (r = 0.30; CI = −0.23 to 0.69; *p* = 0.25), (B) LL (r = 0.21; CI = −0.32 to 0.64; *p* = 0.43), (C) heart rate (r = 0.06; CI = −0.45 to 0.54; *p* = 0.82) and (D) SpO_2_ (r = −0.31; CI = −0.70 to 0.22; *p* = 0.25). Analysis was performed using data from day2 of high altitude exposure. Crosses indicate four participants with highest, squares those with lowest RNFL thickness measurements. Red lines indicate bivariate normal ellipse (*p* = 0.95); blue lines fit simple regression equations; n = 16.

Pearson's correlation analysis revealed no statistically significant correlation between ODE and AMS, when comparing with the four key stereometric parameters (mean RNFL thickness, RNFL cross sectional area, rim volume and maximum contour elevation) that showed significant alteration at altitude with AMS-c, LL, SpO_2_ or HR. Ratios of mean RNFL thickness did not correlate with AMS-c, LL, HR and/or SpO_2_ as shown in Figure 4A–D. Neither did ratios of RNFL cross sectional area, maximum contour elevation and rim volume correlate with AMS-c, LL, HR and/or SpO_2_. For detailed overview, complete data set is presented in Table 4. No correlation was found for ratios of maximum and mean cup depths, which were significantly increased on day2 (data not shown). Pearson's correlation coefficient was calculated to assess a potential correlation of age and the 4 key stereometric parameters on day2 using AMS-c and LL. Both scores correlated significantly with age (AMS-c: r = −0.51; CI = −0.79 to −0.06; *p* = 0.03 and LL: r = −0.63; CI = −0.85 to −0.24; *p*<0.01; n = 18). However, none of the key stereometric parameters correlated with age: mean RNFL thickness (r = −0.09; CI = −0.56 to 0.43; *p* = 0.74), RNFL cross sectional area (r = −0.13; CI = −0.59 to 0.39; *p* = 0.64), rim volume (r = −0.13; CI = −0.59 to 0.39; *p* = 0.63) and maximum contour elevation (r = −0.17; CI = −0.61 to 0.36; *p* = 0.53).

**Table 4:** Pearson's correlation of key stereometric parameters with clinical scores of AMS on day2.

	AMS-c	LL	HR	SpO_2_
**Mean RNFL thickness [mm]**	0.30; −0.23–0.69; 0.25	0.21; −0.32–0.64; 0.43	0.06; −0.45–0.54; 0.82	−0.31; −0.70–0.22; 0.25
**Maximum contour elevation [mm]**	0.40; −0.12–0.75; 0.12	0.24; −0.29–0.66; 0.36	0.33; −0.20–0.70; 0.22	−0.26; −0.67–0.27; 0.33
**RNFL cross sectional area [mm^2^]**	0.32; −0.21–0.71; 0.22	0.23; −0.30–0.65; 0.38	0.03; −0.47–0.52; 0.90	−0.29; −0.69–0.24; 0.28
**Rim volume [mm^3^]**	0.43; −0.08–0.76; 0.09	0.29; −0.24–0.69; 0.27	0.03; −0.47–0.52; 0.90	−0.22; −0.65–0.31; 0.41

Values are presented in the following order as: r, 95% confidence interval (CI), *p*-value.

**Figure 5:**
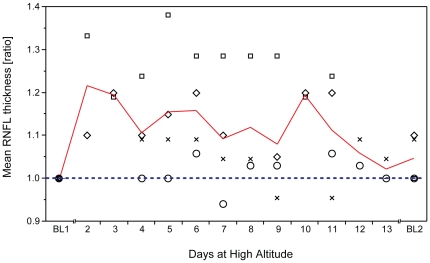
Optic disc edema during long term exposure at high altitude. Optic disc edema (ODE) persists beyond acclimatization. Mean retinal nerve fiber layer (RNFL) thickness ratios in four participants with extended exposure to altitude at CM (4559 m) indicate elevated ratios for RNFL thickness up to day12 of exposure. Blue dashed lines indicate BL1 level; red lines indicate mean; n_day2, 12–13_ = 2, n_day3–7, 9–11_ = 4, n_day8_ = 3.

Changes at the ONH were also monitored for a longer period of high altitude exposure in four participants (investigators) who spent 13 consecutive days at the CM without descending. As shown in Figure 5, the ratio of mean RNFL thickness, which was significantly increased in all participants on day2–4 at high altitude (Figure 3A), showed a comparable increase. Mean ratios remained elevated at altitude up to day10 before decreasing towards and almost reaching baseline levels on day11 to day13. RNFL cross sectional area, rim volume and maximum contour elevation showed matching trends (data not shown).

## Discussion

This study was undertaken to objectively quantify optic disc changes during high altitude exposure and evaluate them within the context of previous reports of high altitude-related ODE. Assessment of the ONH as visible part of the brain is of clinical importance since changes in morphology may provide further insight into the pathophysiology of AMS.

Upon high altitude exposure the majority of participants developed bilateral ODE, i.e. 64% on the day of arrival at CM and 79% on the next morning as described by slitlamp biomicroscopy. Significant ODE was detected objectively using cSLO at high altitude on day2, 3 and 4. No correlation between ODE and AMS at high altitude was found. All findings completely resolved after exposure without detectable residuals at low altitude showing no statistically significant changes between BL1 and BL2, even in volunteers featuring greatest changes at the ONH or in those with highest AMS scores. To the best of our knowledge this is the first study to use state of the art, clinical diagnostic equipment such as a LED-powered slitlamp and HRT3® for detailed quantitative evaluation of optic disc changes.

The incidence of ODE determined clinically by stereoscopic slit lamp biomicroscopy at high altitude was comparable to the data presented by Bosch et al., despite considerable differences between ascent protocol, maximum elevation, exposure time and acclimatization state [10]. Although highly valuable and indispensible in clinical practice, stereoscopic slit lamp biomicroscopy has the disadvantage of being a subjective method for judgment of the optic disc, unable to deliver objective and quantitative data [25], [26]. Since the introduction of cSLO in the HRT1®, ophthalmologists are in the position to objectively determine and quantify ODE, and other alterations of the ONH [27], [28]. The most relevant stereometric parameters for assessing ODE (mean RNFL thickness, RNFL cross sectional area, rim volume and maximum contour elevation) were found to increase significantly during high altitude exposure. Our data indicate the development of ODE as soon as 6 hrs after arrival at the CM (day1). On day2 and subsequent days 3 and 4, all key stereometric parameters were significantly increased (*p*<0.05). In the four participants who stayed at the CM for 13 consecutive days, all stereometric parameters remained elevated until day10, while AMS-c and LL scores already started to decrease from day2 and were zero from day4 onwards.

A vast majority of clinical changes at the ONH consisted of evident edema with only very few equivocal changes. All discs judged as edematous would have prompted physicians to order subsequent testing in otherwise healthy patients. While all stereometric parameters showed statistically significant changes supporting the notion of altitude related ODE, absolute changes ranged from 5–8% (Table 3). The magnitude of changes fits well with reports of disc swelling in patients with optic neuritis while studies in arteritic or non-arteritic ischemic optic neuropathy found larger changes at the ONH [29], [30]. All alterations of the optic disc were completely reversible upon return to low altitude (Table S1) with no detectable sequels of ODE at BL2 even in participants who suffered from severe AMS. This makes any chronic effects unlikely. While long-term observations are needed to definitively exclude any residual structural and functional changes, it seems unlikely that acute short-term exposure to an altitude of 4559 m is able to induce permanent damage to the optic nerve considering the large number of mountaineers reaching this or even much higher altitudes with no reported long term pathology.

As anticipated, our ascent profile induced AMS in roughly 50% of our participants fitting well with the predicted 30% to 60% range for this kind of ascent profile depending on individual susceptibility [31]. To objectively and quantitatively test for a correlation between ODE and AMS-c, LL, HR, SpO_2_ or age, we used HRT3® readings acquired on the first morning at the CM (day2) because AMS related scores reached peak values at that time point (Table 1), which is in line with existing literature [12]. On day1, exhaustion would have been suspected to skew clinical scores and on day3 the AMS related scores already decreased dramatically, while ODE parameters remained elevated throughout all days at high altitude (Figure 3). However, the analysis yielded no significant correlation of key stereometric parameters with AMS-c or LL. The highest, yet still non-significant correlation was observed for AMS-c *vs.* rim volume (r = 0.43; CI =  −0.08 to 0.77; *p* = 0.09) and again for LL *vs.* rim volume (r = 0.30; CI = −0.24 to 0.69; *p* = 0.23). Previous studies have suggested several other ocular changes under hypoxia and/or high altitude exposure to correlate with AMS as measured by AMS-c and LL, among them optic disc swelling, increased optic nerve sheath diameter, increased corneal thickness and increased retinal capillary blood flow [10], [15], [32], [33]. In contrast, pupil dynamics and intra ocular pressure have been found not to correlate with symptoms of AMS [34], [35]. While our data showed no relevant correlation between ODE and AMS, and ODE and resting HR, O'Connor et al. have found a correlation between severity of AMS and HR [36]. Our data supports previous data suggesting a lower incidence of AMS in older people with a moderate but significant Pearson's coefficient of r = −0.51 in our participants [37], [38]. However, none of the key stereometric HRT3® parameters correlated significantly with age. Therefore we hypothesize that AMS may correlate with age, but independently of ODE. Support against a correlation of ONH changes and AMS can also be drawn from the independent development of parameters of the ONH and AMS scores with HRT3® stereometric parameters remaining elevated at high altitude over 10 days and AMS scores being 0 from day4 onward (Figure 5).

The pathophysiology of AMS has –despite considerable interest and research efforts in the last decades– remained a conundrum and cause of debate in high altitude physiology. It has been proposed that AMS is a subclinical form of early HACE, sharing a common pathophysiological background [39]. Extracellular vasogenic edema caused by disruption of the blood-brain-barrier (BBB) leading to raised intracranial pressure and mechanical vascular leakage has been suggested to play a key role in the development of severe AMS and HACE [11], [40]. However, a study conducted by Bailey et al. detected no evidence for BBB dysfunction, nor conclusive evidence for a significantly raised intracranial pressure with only minimal increase in overall brain volume regardless of AMS symptoms [16], which is in line with other studies [41], [42]. Kallenberg et al. suggested that extracellular vasogenic edema contributes to generalized brain edema independent of AMS, while cytotoxic intracellular edema may correlate with AMS [43]. Thus, it has been suggested that mild brain swelling during sustained periods of hypoxia or high altitude exposure occurs, but does not correlate with AMS.

Previous studies have consistently confirmed the existence of ODE at high altitude albeit with differences to as to its incidence. This might well be due to different ascent profiles or duration of altitude exposure. This is why we chose a well-established ascent profile for our study with the primary goal to study ocular changes in regard to AMS at high altitude [44], [45]. One of the major strengths of our study was the nature of our study design. We used the research facility of the CM, which provides outstanding research conditions at high altitude ensuring standardized procedures. Objective, quantitative outcome measures obtained by the same experienced investigators using state of the art equipment were used. By testing for intraindividual differences over time, each participant acted as its own control solving the problem of interindividual differences in cross sectional analyses. This is important as such a field study provides limited possibilities to increase the number of participants.

Our clinical funduscopic examinations and quantitative HRT3® values constitute evidence for symmetric bilateral ODE occurring in the majority of persons exposed to high altitude. As a mild increase of brain volume during hypoxia exposure independent of elevated lumbar pressure has been reported [16], we speculate that bilateral ODE caused by altitude exposure is not caused by increased ICP. Thus ODE may not be termed high altitude related papilledema as this entity is defined by increased ICP. ODE is also described in disturbed venous outflow when capillary venous pressure in the retinal, ciliar or combined system of the ophthalmic artery is increased due to thrombosis, cardiac insufficiency, etc. [46]. Since increased diameters of retinal veins have been well described and are a hallmark of high altitude retinopathy, it seems plausible that this mechanism may be responsible for the ONH changes observed at high altitude. Cytotoxic intracellular edema as described for the brain by MRI studies at normobaric hypoxia [43] may also contribute to ODE as a result of reduced axonal transport [17], [47].

In conclusion our present findings indicate that ODE occurs during hypoxia exposure at high altitude, may persist over time despite further acclimatization and is fully reversible after descent to lower altitudes. However, in contrast to a previous study we did not find a correlation between AMS and ODE. Therefore, we suggest that ODE occurs independently of AMS during high altitude exposure.

## Supporting Information

Table S1 **Overview of HRT3® readings between BL1 and BL2.**
(DOCX)
