# Supplementary material for: Quantification of Optic Disc Edema during Exposure to High Altitude Shows No Correlation to Acute Mountain Sickness
Source: PLoS One. 2011 Nov 1;6(11):e27022. doi: 10.1371/journal.pone.0027022 (PMC3206056; doi:10.1371/journal.pone.0027022)
Supplement: Table S1 — Overview of HRT3® readings between BL1 and BL2. (DOCX) [file pone.0027022.s001.docx]

| **Supplemetal Table 1. Overview of HRT 3 readings between BL1 and BL2** | | | | |
| --- | --- | --- | --- | --- |
|  | **change** | **95% CI ↓** | **95% CI ↑** | ***p*-value** |
| average variability (SD) [mm] | 0.001 | -0.004 | 0.005 | 0.80 |
| cup area [mm^2^] | 0.004 | -0.174 | 0.183 | 0.96 |
| cup volume [mm^3^] | 0.001 | -0.050 | 0.051 | 0.98 |
| disc area [mm^2^] | 0.001 | -0.362 | 0.363 | 0.99 |
| maximum contour elevation [mm] | 0.010 | -0.039 | 0.067 | 0.60 |
| maximum cup depth [mm] | 0.006 | -0.093 | 0.105 | 0.90 |
| mean cup depth [mm] | 0.002 | -0.035 | 0.038 | 0.93 |
| mean RNFL thickness [mm] | 0.003 | -0.039 | 0.044 | 0.90 |
| rim area [mm^2^] | 0.006 | -0.225 | 0.237 | 0.96 |
| rim volume [mm^3^] | 0.004 | -0.079 | 0.088 | 0.92 |
| RNFL cross sectional area [mm^2^] | 0.001 | -0.202 | 0.203 | 0.99 |
| vertical cup/disk ratio | 0.010 | -0.100 | 0.121 | 0.85 |
